# Multi-disciplinary diagnosis and management of verrucous venous malformation of the right knee: a case report

**DOI:** 10.3389/fradi.2025.1686404

**Published:** 2026-01-13

**Authors:** Varun H, Bhushan Madke, Prerit Sharma, Adarshlata Singh, Anurag Mittal, Vedashree Vedprakash Tiwari

**Affiliations:** 1 Department of Dermatology, Venereology and Leprosy, Datta Meghe Institute of Higher Education and Research, Jawaharlal Nehru Medical College, Wardha, Maharashtra, India; 2 Department of Interventional Radiology, Datta Meghe Institute of Higher Education and Research, Jawaharlal Nehru Medical College, Wardha, Maharashtra, India; 3 Department of Radiodiagnosis, Mahadevappa Rampure Medical College, Kalaburgi, Karnataka, India

**Keywords:** vascular malformations, venous malformation, dermoscopy, magnetic resonance imaging, dermatology, bleomycin, sclerotherapy, interventional radiology

## Abstract

Verrucous Venous Malformations (VVMs) are a rare subtype of Congenital Vascular Malformations (CVMs) characterised by a hyperkeratotic, verrucous surface. We present the case of a ten-year-old male with a VVM localised to the right knee, which presented as a gradually enlarging, asymptomatic lesion since birth. A comprehensive, multi-modality diagnostic workup was performed, including thorough clinical evaluation, dermoscopy, radiologic imaging (Plain radiograph, colour Doppler ultrasonography and magnetic resonance imaging) and histopathological analysis with hematoxylin and eosin staining, along with immunohistochemical staining for CD-34. The lesion exhibited characteristic features consistent with VVM. The patient was managed by percutaneous sclerotherapy to reduce lesion size. This case highlights the importance of a multidisciplinary strategy in the diagnosis and management of VVMs to improve clinical outcomes.

## Highlights

• VVM is a rare CVM characterised by hyperkeratotic, verrucous skin changes.
• Comprehensive workup including dermoscopy, radiologic imaging, histopathology and IHC helped accurate diagnosis and exclusion of mimics.
• 1st sitting of sclerotherapy included percutaneous bleomycin injection under USG and fluoroscopic guidance, achieving a significant reduction in lesion size and improved appearance.
• Multidisciplinary collaboration between dermatology, radiology, pathology and interventional radiology was critical for diagnosis and management.
• Early recognition and targeted therapy can improve outcomes and reduce the need for more complicated and invasive interventions.

## Introduction

Vascular anomalies are common soft tissue abnormalities that are classified into proliferating vascular tumours and Congenital Vascular Malformations (CVM) (1). Venous malformations are amongst the most common types of CVM. They typically present as asymptomatic lesions at birth, progressively enlarging over time, causing significant pain and discomfort (2). Verrucous Venous Malformation (VVM) is a rare subtype of vascular malformation characterised by reactive cutaneous changes such as papillomatosis, acanthosis, and hyperkeratosis. Clinically, VVMs present as solitary or multiple reddish-brown hyperkeratotic papules or plaques (3). The diagnosis and management of vascular malformations pose considerable challenges, often requiring a multidisciplinary approach (4).

Herein, we present a case of a young male patient with a VVM localised to the right knee, initially presenting as a slow-growing swelling circumferentially involving the knee joint. Our report details the clinical, dermoscopic, histopathologic and radiological features of VVM. The lesion was managed with sclerotherapy, which led to a significant reduction in lesion size.

## Case description

A 10-year-old male presented to the dermatology outpatient clinic with swelling and a raised, rough lesion on his right knee since birth. Per parental history, the lesion originated as a small red-brown spot at birth and demonstrated gradual expansion over the subsequent decade, accompanied by disproportionate circumferential enlargement of the right knee and thigh compared to the contralateral limb. The patient reported recurrent bleeding episodes after minimal physical trauma, consistently controlled upon applying local pressure. He also complained of occasional, dull-aching pain that would spontaneously resolve or with self-administered non-steroidal anti-inflammatory drugs (NSAIDs). No restriction in knee joint mobility was observed.

**Figure 1:**
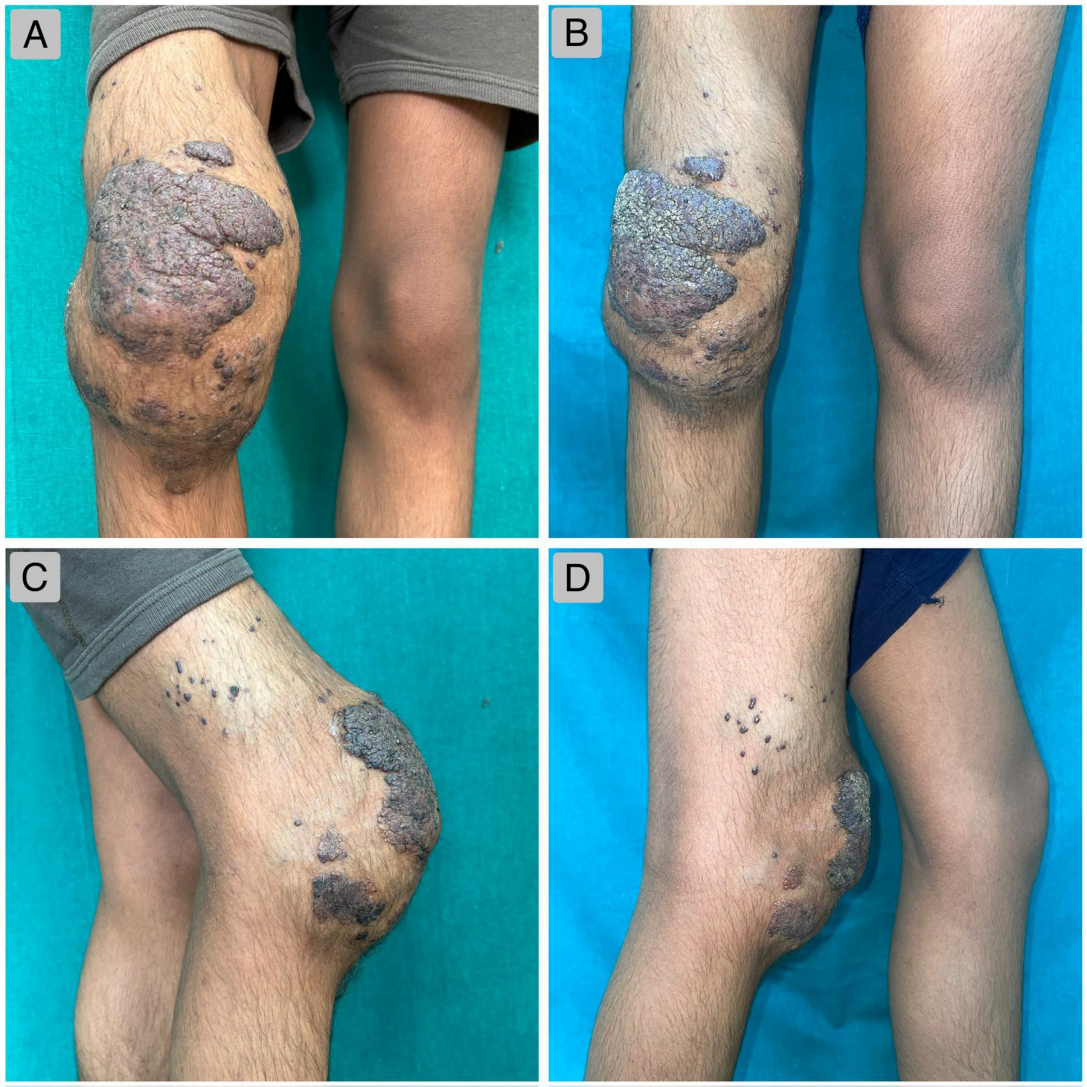
**(A)** (anterior)—Pre-embolisation clinical image demonstrating a well-defined, hyperpigmented plaque with a verrucous surface on the anterior aspect of the knee, accompanied by multiple papules with a similar verrucous surface around the joint. **(B)** (Anterior)—Clinical image one month post-embolisation, showing a significant reduction in the verrucous surface of the lesion. **(C)** (Lateral)—Pre-embolisation clinical image of the lateral aspect of the knee joint, demonstrating marked enlargement of the knee along with bluish vascular papules encircling the knee joint. **(D)** (Lateral)—post-embolisation clinical image showing a significant reduction in the girth of the right knee.

**Figure 2:**
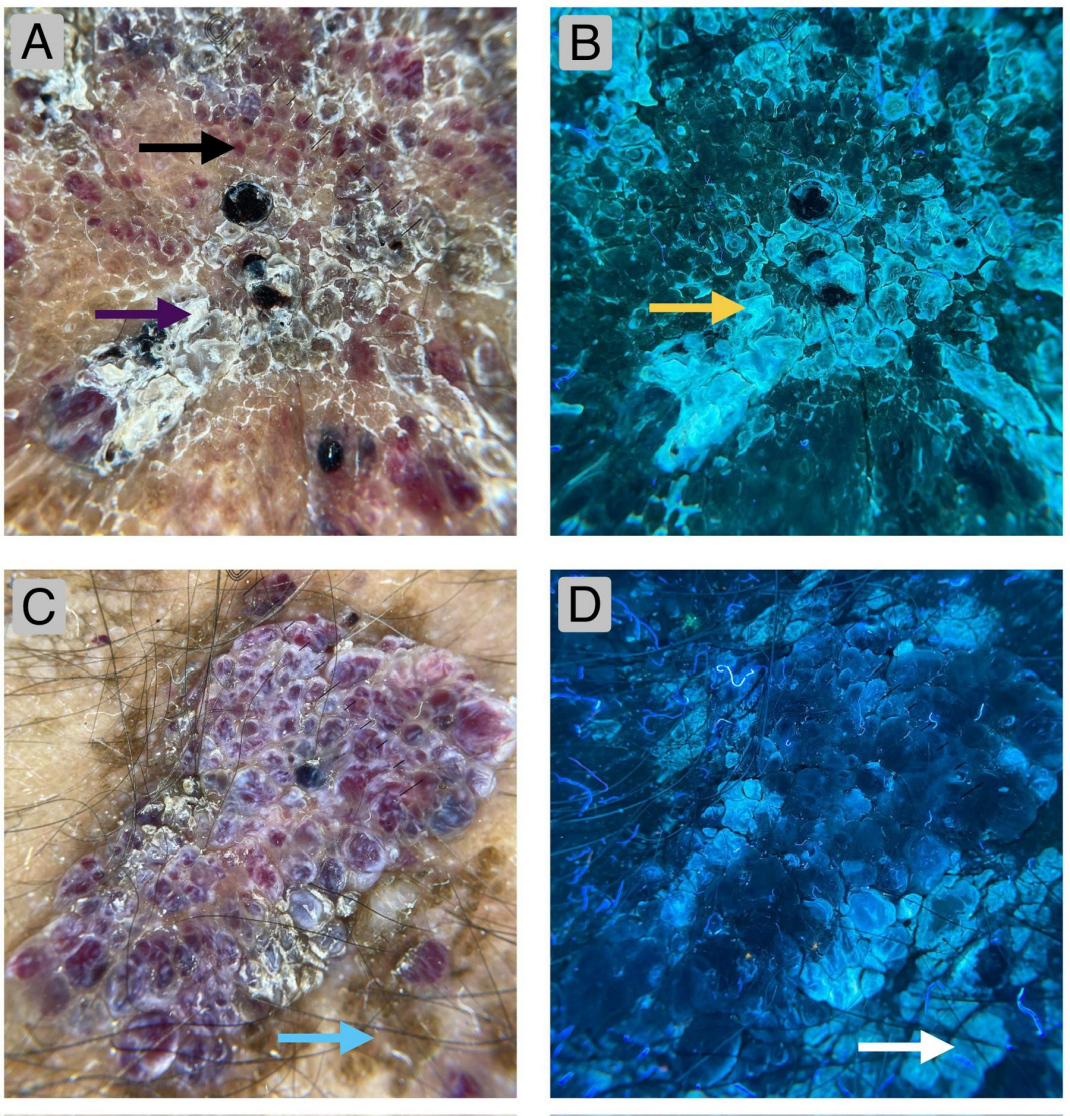
**(A)**—dermoscopic image (DermLite DL5, cross-polarised light coupled with iPhone 12 camera) demonstrating reddish to dark blue lacunae (black arrow) accompanied by prominent hyperkeratosis (purple arrows). **(B)**—UVFD image of the same field (DermLite DL5, Wood-Mode 365 nm, coupled with iPhone 12 camera) showing white fluorescence of the hyperkeratotic areas (yellow arrow). **(C)**—Dermoscopic image (DermLite DL5, cross-polarised light coupled with iPhone 12 camera) demonstrating areas of initial hyperkeratosis, minimally seen via cross-polarised light (blue arrow). **(D)**—UVFD image of the same field (DermLite DL5, Wood-Mode 365 nm, coupled with iPhone 12 camera) depicting fluorescence of keratin in hyperkeratotic areas not evident in cross-polarised light (white arrow).

On examination, multiple red-brown papules and a solitary verrucous plaque measuring 10 × 15 cm with well-defined purplish borders were observed on the anterior aspect of the right knee joint (Figures 1A,C). The girth of the right knee was seven centimetres greater than that of the contralateral side. The lesion was soft and compressible on palpation and demonstrated a low-pitched bruit on auscultation. Contact dermoscopy under cross-polarised light revealed multiple reddish-to-dark blue lacunae, indicative of dilated vascular channels within a bluish background, along with prominent central hyperkeratosis (Figure 2A). The corresponding UVFD image demonstrated bright white fluorescence within the hyperkeratotic areas (Figure 2B). A second representative field examined under cross-polarised dermoscopy showed early hyperkeratotic changes with scattered vascular lacunae (Figure 2C), and the matching UVFD image again revealed white fluorescence within the keratinous components (Figure 2D).

Based on clinical and dermoscopic findings, we established a provisional diagnosis of VVM with differential diagnoses including: angiokeratoma circumscriptum, lymphatic malformations, arteriovenous malformations, Kaposiform haemangioendothelioma and verrucous epidermal nevus. Notably, we excluded the outdated term “verrucous haemangioma” as per the International Society for the Study of Vascular Anomalies (ISSVA) classification. A comprehensive detailing of differential diagnosis and exclusion rationales is listed in Table 1.

**Table 1:** Summarising differential diagnosis.

Diagnosis	Distinguishing Features	Exclusion Rationale (Case-Specific Findings)
Angiokeratoma circumscriptum	- Superficial dermis only - Dermoscopy: central whitish veil surrounding, and over red-blue lacunae (26)	- Deep subcutaneous extension on MRI - Dermoscopy: Absent whitish veil; demonstrated dark blue and purple lacunae with hyperkeratosis.
Lymphatic Malformation	- Fluid-fluid levels on MRI (when complicated with haemorrhage) (12)	- Enhancing vascular channels seen on MRI - Absence of cystic fluid-filled spaces.
Extracranial arterio-venous malformations	- Palpable thrill on palpation - Doppler: High-flow arterial waveforms on doppler (14).	- Soft, compressible mass without thrill. - Colour Doppler: Slow-flow venous vessels only
KHE	- Thrombocytopenia (in 70–78% cases) - Histopathology: Spindle cells creating vascular slits (15, 16).	- Normal platelet count - Histopathology: Multiple dilated venules (no spindle cells)
Verrucous Epidermal Naevus	- Blaschkoid distribution (27, 28). - Histopathology: Acanthosis, hyperkeratosis, papillomatosis without vascular channeles.	- Circumferentially knee involvement (non-Blaschkoid) - Histopathology: Vascular channels.
Seborrheic keratosis	- “Stuck-on”, waxy-brown papules and plaques. - Late onset >40 years - Dermoscopy: Milia like cysts and comedo-like openings - Histopathology: Acanthosis keratin pseudocysts. (29, 30)	- Congenital onset - Dermoscopy: Vascular lacunae - Histopathology: RBC-filled vascular channeles.

**Figure 3:**
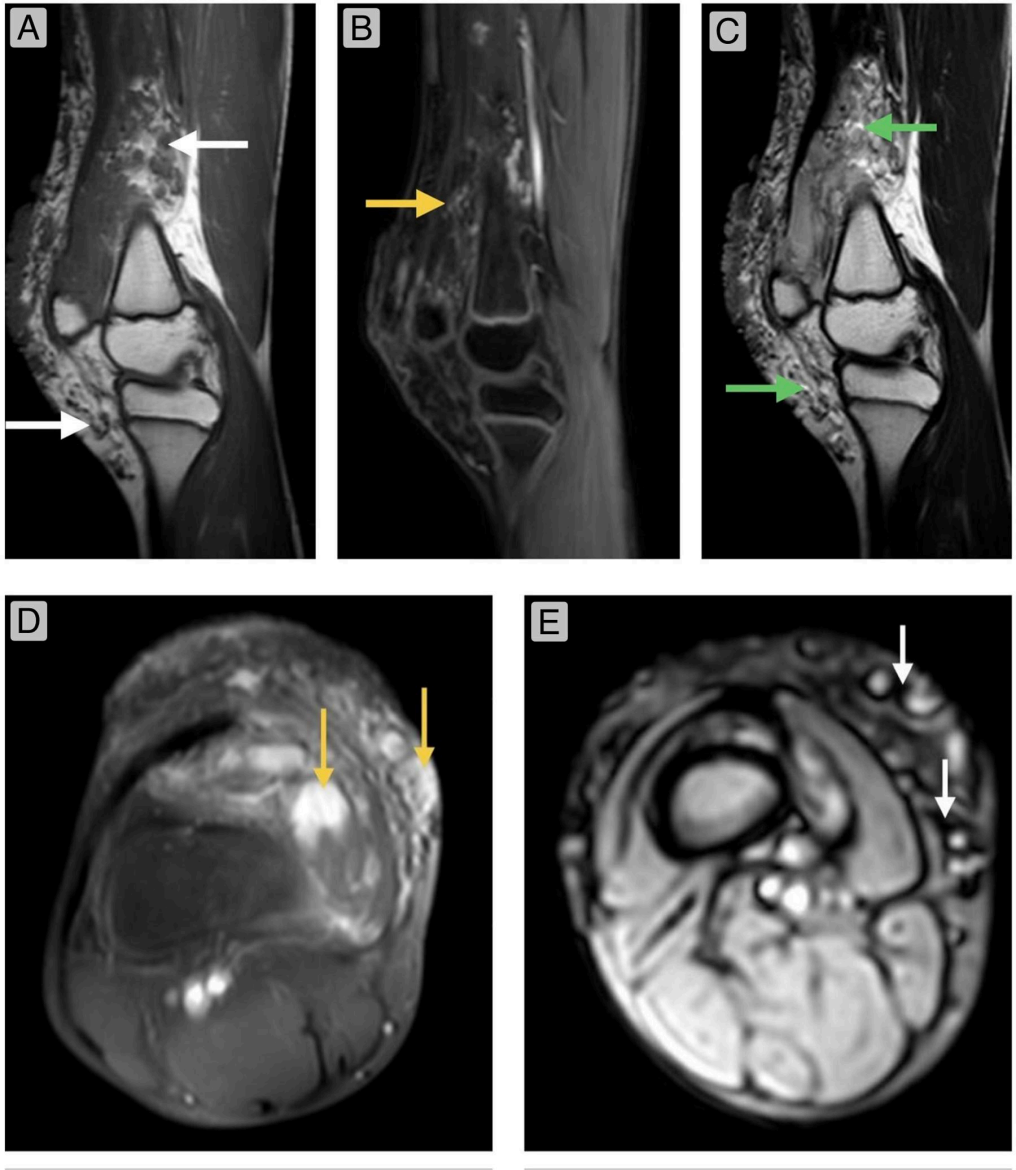
**(A)**—sagittal T1-weighted turbo spin echo (TSE) image demonstrates typical hypointense tubular structures (serpentine vessels) (white arrows) within a hyperintense stroma in the subcutaneous plane, showing minimal extension into the rectus femoris and vastus lateralis. **(B)**—Sagittal T1-weighted image (post-gadolinium contrast) demonstrating slow and gradual but vivid, heterogeneous enhancement of the mass (indicating slow-flow vascular spaces) (Yellow arrow) with few non-enhancing foci. **(C)**—Sagittal T2-weighted TSE image demonstrating high signal intensities (hyperintense lobules with septations) (green arrow). **(D)**—Axial T1-weighted TSE image (post gadolinium contrast) demonstrating intense, heterogeneous enhancement (yellow arrows). **(E)**—Axial GRE image demonstrating low signal intensities (white arrows).

A plain radiograph of the right knee showed an ill-defined, lobulated soft tissue lesion with a few subtle, rounded specks of radio-opacities suggestive of phleboliths (Supplementary Image 1) in the suprapatellar and infrapatellar regions with no joint space involvement. Ultrasonography examination demonstrated a heterogeneous mass with multiple compressible anechoic channels predominantly demonstrating a low flow on colour Doppler and a few hyperechoic foci with posterior acoustic shadowing in the subcutaneous plane, findings consistent with slow-flow venous malformation. Magnetic resonance imaging (MRI) demonstrated a lobulated multiseptated mass involving the subcutaneous plane, showing a small intramuscular extension. It exhibited low to intermediate signal intensity on T1-weighted images and high signal on T2-weighted and Proton Density Fat Saturated (PDFS) sequences. The lesion also demonstrated several scattered foci of low signal intensities on gradient-recalled echo (GRE) sequences. In contrast-enhanced MRI with gadolinium, there was slow and gradual yet vivid heterogeneous enhancement of the mass with a few non-enhancing foci (Figure 3).

**Figure 4:**
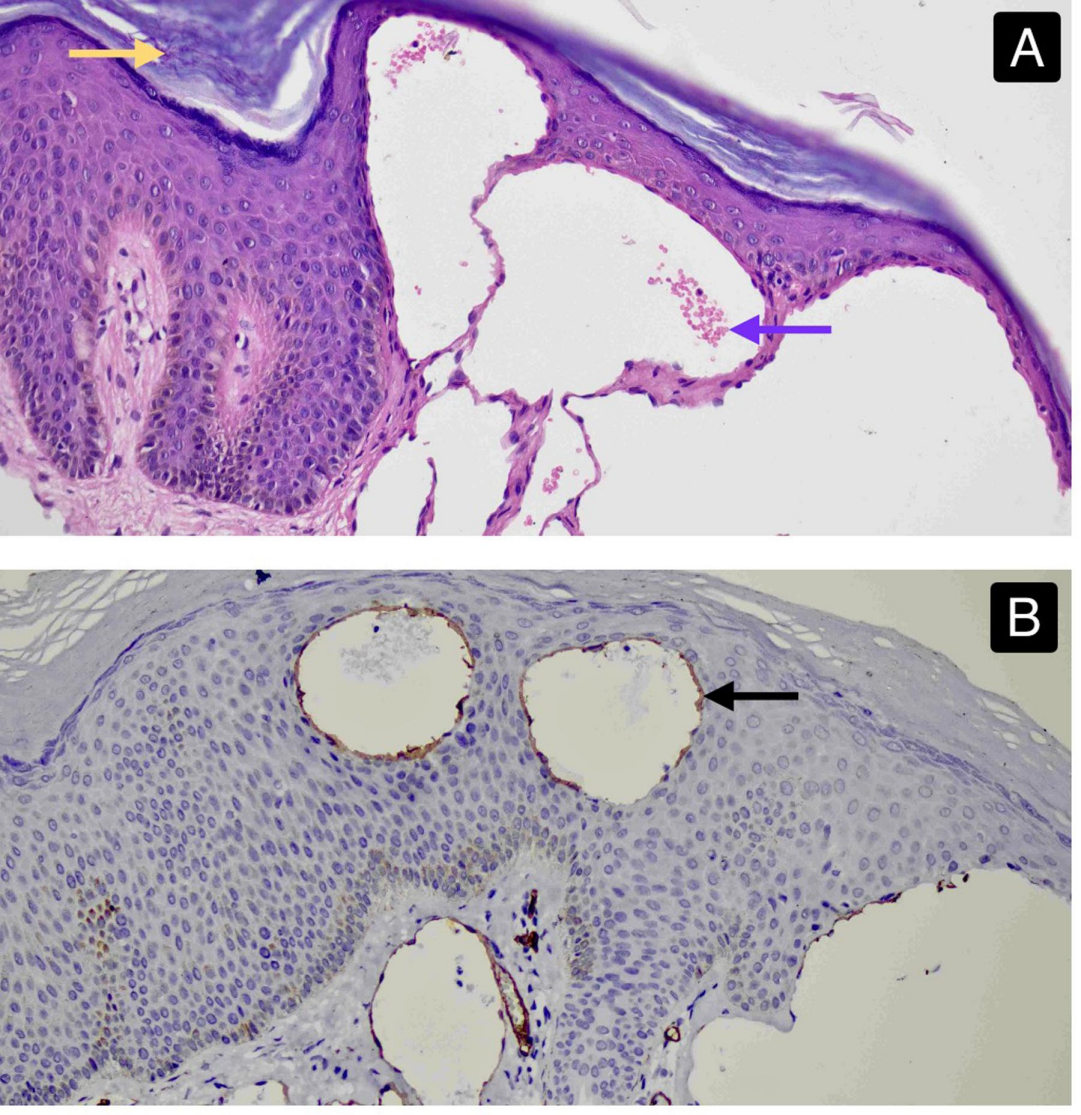
**(A)**—histopathological examination of a punch biopsy of a papule from the right knee joint stained with haematoxylin and eosin, demonstrating hyperkeratosis (yellow arrow) and dilated vascular elements containing red blood cells (purple arrow) (10x ocular lens coupled with a 20x objective lens producing an effective magnification of 200x). **(B)**—Histopathological examination of a punch biopsy of a papule from the right knee joint with immunohistochemical staining, revealing deep brown staining of the vascular endothelium with CD-34 (Black arrow) (10x ocular lens coupled with a 20x objective lens producing an effective magnification of 200x). NOTE—This case report was prepared following the CARE Guidelines (40).

A punch biopsy of a papule was performed, and histopathological examination with haematoxylin and eosin (H&E) staining revealed dilated vascular channels containing erythrocytes within the epidermis and papillary dermis (Figure 4A). Immunohistochemical (IHC) staining for CD34 demonstrated diffuse brown staining of the vascular endothelium, confirming the vascular nature of the lesion (Figure 4B). All routine blood parameters, including coagulation profile, D-dimer levels and fibrinogen levels, were within normal limits.

Based on the clinical evaluation, dermoscopic and radiological findings, and histopathological confirmation via biopsy, a diagnosis of VVM was established. The patient was subsequently referred to Interventional radiology (IR) for percutaneous sclerotherapy of the malformed vasculature. Lower limb angiography was first performed to exclude arterial components (Supplementary Video 1), confirming the absence of feeding arteries. To streamline care, the operating interventional radiologist proceeded with immediate sclerotherapy during the same session (Supplementary Video 2).

Under combined Doppler ultrasound and fluoroscopic guidance:

• A 22-gauge butterfly needle was advanced into the venous channels until a venous outflow was detected.
• Deeper lesions were cannulated using a 22-gauge spinal needle under USG guidance, with blood aspiration verifying intravascular placement.
• Iopromide contrast injection under fluoroscopy delineated lesion dimensions (Supplementary Image 2).
• 15 mg of reconstituted bleomycin was infiltrated into the venous channels using a double-needle technique, with the second needle used as a vent permitting excess agent egress.
• The injection was stopped when clear fluid emerged from the vent needle.

Post-procedurally, a compression bandage was applied for 48 h. The recovery period was uneventful.

One month post-sclerotherapy, the right knee exhibited a significant reduction in girth and a marked improvement in the verrucous appearance of the lesion (Figures 1B–D). Repeat MRI confirmed a substantial reduction in the vascular channels (Supplementary Image 3). At the time of writing, the patient has undergone a single session of bleomycin sclerotherapy and is scheduled for follow-up; however, he has not yet returned for his subsequent evaluation. Additional sessions will be undertaken as required, based on clinical response, MRI findings, and persistence of venous channels, as part of a planned staged management approach. A chronological overview of the patient's presentation, diagnostic workup, treatment, and short-term outcome is summarised in Table 2.

**Table 2:** Timeline of events pertaining to case report data.

Sequential events of the case	Interpretation with relevant data
Case presentation (Symptoms and brief history) (June 29th, 2,025)	A 10-year-old male presented with a gradually enlarging, reddish-brown swelling since birth associated with occasional, controllable bleeding following minimal trauma, with no restriction of movement.
Clinical examination (June 29th, 2025	A Single, well-defined, hyperpigmented plaque with a hyperkeratotic, verrucous surface was observed over his right knee joint, surrounded by multiple well-defined, bluish papules (Figures 1A,C)). Dermoscopic examination revealed multiple reddish-to-blue vascular elements with prominent hyperkeratosis (Figures 2A,C). UVFD demonstrated enhanced visualisation of the keratin through white fluorescence, allowing the detection of hyperkeratotic areas not visualised under polarised light (Figures 2B,D).
Radiological investigations (June 30th, 2025)	Plain radiograph—ill-defined soft tissue mass was observed in the suprapatellar and infrapatellar regions with no involvement of joint space.
	Colour Doppler ultrasound—An ill-defined vascular lesion predominantly located in the subcutaneous tissue, exhibiting internal vascularity with a predominance of slow-flow vessels.
	MRI—The lesion demonstrated relatively well-defined, lobulated margins, appearing hypointense on T1-weighted images and hyperintense on T2-weighted and PDFS sequences. It exhibited restricted diffusion and multiple scattered foci of low signal intensity on GRE. Post-contrast imaging revealed substantial but heterogeneous enhancement (Figures 3A–E).
Punch biopsy (Taken on July 1st^,^ reported on July 10th, 2025)	H&E staining revealed dilated vascular channels within the reticular dermis (Figure 4A). IHC staining for CD34 showed brown staining of the endothelial lining, confirming vascular origin (Figure 4B).
Treatment	- 1st sitting of percutaneous sclerotherapy (July 20th, 2025) resulted in a significant reduction in plaque size (Figures 1B,D). - Further sclerotherapy sessions planned.
Outcome	Significant reduction in lesion size was seen twenty days after the first session of sclerotherapy. The patient is scheduled for follow-up.

The diagnostic and therapeutic course described above illustrates the complex, multisystem nature of VVM. In the following section, we situate these findings within the current evidence base, highlighting key diagnostic features and management considerations related to VVM.

## Discussion

CVMs are present at birth in approximately 10% of newborns. In contrast, VVMs are rare. VVM is a slow-growing congenital malformation, typically located in the lower extremities, sometimes following linear or Blaschkoid patterns (5). It is characterised by a progressively hyperkeratotic, verrucous surface (6). Immunoprofiling reveals no involution for WT1 and GLUT-1, in contrast to vascular tumours, which are positive for these markers (7, 8). Accordingly, the ISSVA categorises VVM as a venous malformation rather than a vascular tumour. Recent studies by Couto JA et al. identified a somatic MAP3K3 mutation associated with VVM (9).

Clinically, angiokeratoma circumscriptum is an important differential diagnosis for VVM, with other possibilities including lymphatic malformations, arteriovenous malformations, Kaposiform hemangioendothelioma (KHE), verrucous epidermal nevus, pigmented basal cell carcinoma, and seborrheic keratosis (Table 1). In our case, angiokeratoma circumscriptum was excluded dermoscopically due to the absence of “white veil” and perilesional erythema, and by its typical confinement to the superficial papillary dermis rather than the deeper dermis and subcutis seen in VVM (10). Lymphatic malformations were ruled out based on histology showing erythrocyte-filled vascular channels and MRI findings of partially open venous pouches with blooming artefacts or phleboliths on GRE, unlike the large multiloculated cystic spaces and fluid–fluid levels characteristic of lymphatic malformations (11–13). Although simple venous malformations may appear similar radiologically, the presence of hyperkeratosis and reddish-blue lacunae on dermoscopy supported VVM. Arteriovenous malformations were excluded by Doppler ultrasound, demonstrating exclusively slow-flow venous channels with no arterial components or arterialised venous waveforms (14). KHE was ruled out due to normal platelet counts—thrombocytopenia being common in KHE due to Kasabach-Merritt phenomenon (15, 16)—absence of rapid progression or bony involvement, and histopathology showing dilated venules rather than spindle-cell proliferation forming slit-like vascular channels.

Although historically termed “verrucous haemangioma”, this term is misleading and should not be used, because “haemangioma” implies a proliferative pathology (vascular tumour). ISSVA classification correctly designates these lesions as venous malformations, consistent with their congenital onset, slow progression, and local tissue hypertrophy (17).

Accurate diagnosis of VVM requires a multi-modal approach–clinical examination, which identifies bluish plaques with prominent hyperkeratosis, dermoscopy that helps visualise multiple dark-blue lacunae with keratin flakes, USG with colour Doppler helps to assess vascular flow patterns, multisequence MRI to exclude VVM mimics (angiokeratoma, lymphatic/arterio-venous malformations), histopathology (Deep biopsy including the subcutis) to look for large dermal-venule-like channels containing erythrocytes ruling out capillary and lymphatic malformations. IHC can further confirm the vascular origin by using CD34 positivity and GLUT-1 negativity to rule out haemangiomas (14). Additionally, for equivocal cases, an extended IHC panel consisting of podoplanin and PROX1 can be used to rule out other vascular malformations, while alpha-SMA IHC staining will further help to show the venous malformation's characteristics (18). Emerging techniques such as cell-free DNA testing can detect somatic MAP3K3 mutations (19). *(In this case Advanced IHC studies, such as GLUT-1, were not performed due to resource limitations.)*

VVM frequently extends into the deeper dermis and subcutaneous tissue, making complete surgical resection challenging and associated with a high recurrence rate, with documented literature suggesting a recurrence rate of about 17.1% post-surgical resection of diffuse and infiltrative vascular malformations (20, 21). Oral sirolimus has shown promising results in a retrospective cohort study involving ten patients with VVM (22). A multidisciplinary diagnostic and therapeutic approach is essential. Dermoscopic evaluation and radiological investigations–including Doppler ultrasonography and MRI–are invaluable in precisely delineating the lesion. This detailed assessment can guide the treatment decisions, such as reducing the vascular channels with sclerotherapy prior to surgical resection, which may lead to improved outcomes. Sclerotherapy is the gold standard approach for reducing lesion size through targeted obliteration of vascular channels (23).

VVMs can extend into deep anatomical structures and can cause significant comorbidities, including consumption coagulopathies. Surgical extension is often challenging due to deep tissue involvement and carries a high recurrence rate. Early diagnosis is therefore crucial to prevent deeper tissue involvement and to facilitate conservative interventions and prevent complications.

In this case, bleomycin was selected as the sclerosant of choice because it provides deeper stromal penetration and carries a lower risk of local tissue necrosis compared with detergent sclerosants such as sodium tetradecyl sulphate (STS). Evidence from a recent systematic review and meta-analysis by De Maria et al. demonstrated that STS had the lowest complete cure rate among commonly used agents (55.5%; 95% CI: 36.1–74.9%), suggesting comparatively reduced efficacy, particularly in complex or deeper venous malformations (24). Considering this lower response rate, together with deeper, multiseptated architecture of our patient's lesion on MRI, bleomycin was deemed more appropriate due to its established efficacy, safety, and reliable penetration in slow-flow venous malformations.

While newer modalities like Bleomycin Electro-Sclerotherapy (BEST) show promise–requiring significantly lower doses of bleomycin and demonstrating superior efficacy with reduced recurrence compared to conventional sclerotherapy–this technique efficiently triggers endothelial apoptosis through electroporation-induced drug uptake. However, BEST needs specialised equipment and general anaesthesia. Given our centre's rural location and resource constraints, we pursued conventional sclerotherapy rather than BEST (25). The currently available medical, interventional, and surgical treatment modalities for VVM, along with their advantages and limitations, are summarised in Table 3.

**Table 3:** Summarising the current treatment modalities for VVM.

Modality	Advantages	Disadvantages
Sclerotherapy (STS)	- Minimally invasive - Effective for superficial channels - Low cost, daycare procedure (31) - Gold standard procedure for reducing lesion size (32).	- Can lead to skin necrosis and ulceration (33). - Requires multiple sessions for significant effects. - Limited efficacy for large and deep lesions.
Sclerotherapy with intra-lesional bleomycin	- Penetrates deeper in comparison to STS (31) - Lower side effect profile than other sclerosants (34)	- Risk of pulmonary fibrosis (higher risk if cumulative dose >400 mg or single dose >30 mg) (Cumulative dose monitoring required) - More effective in lymphatic malformations than venous malformations (23). - Rare reports of limb necrosis after sclerotherapy (general risk of sclerotherapy) (35).
Bleomycin Electro-Sclerotherapy (BEST)	- Superior efficacy in comparison with conventional sclerotherapy - Lower dose of bleomycin required - Enhanced cellular uptake via electroporation (25)	- Specialized equipment needed - General anesthesia required.
Systemic sirolimus (mTOR inhibitor)	- Recently published data has good evidence that suggests good control in diffuse and unresectable VVM. - Prevents progression of growth (36, 37).	- Systemic toxicity (mucositis, pancytopenias and immunosuppression), requires therapeutic drug monitoring (33). - Off-label use - Long-term use required (>2 years)
Topical sirolimus	- Minimal systemic absorption, - Can be suited for pediatric age groups	- Variable efficacy, might not be suited for large lesions (38).
Laser therapy (pulse dye laser)	- Minimally invasive - Effective for small superficial vascular lesions	- Ineffective for large lesions, does not penetrate deep (38). - Causes local skin side-effects such as burns and post-inflammatory hyperpigmentation. - More effective for surface telangiectasias than deep venous malformations. - Adjunct therapy, must be combined with other modalities.
Surgical excision	- Curative potential - Immediate relief from large lesions. - Single-sitting procedure	- High risk of intraoperative blood-loss - Potential functional deficits - Nerve injury, autonomic denervation dermatitis can result at surgical incision site - 17.1% risk of recurrence in large lesions with incomplete margins (20).
Endoscopic Resection	- Minimally invasive - Low chance of recurrence in subcutaneous variants - Superior cosmetic outcome	- Technically complex, long learning curve. - Limited to subcutaneous lesions - Risk of skin necrosis.
Endovenous laser ablation	- Minimally invasive - Minimal to no local side-effects - Precise targeting	- More effective in lesions with intact veins (e.g., Draining vein or varicose veins) (39)

## Conclusion

This case underscores the rarity and diagnostic complexity of VVM, highlighting the value of comprehensive, multi-modality workups—including dermoscopy, advanced imaging, and histopathology with IHC—to establish a final diagnosis. In our case, percutaneous sclerotherapy with bleomycin achieved a significant reduction in lesion size and improvement in clinical appearance, demonstrating its effectiveness as a minimally invasive treatment option, especially in resource-limited settings. A multidisciplinary approach remains critical for optimising outcomes and minimising complications, and careful long-term follow-up is warranted to monitor for recurrence or progression.

## Patient perspective

As parents, we were deeply concerned when we noticed our son's knee swelling and skin changes worsening over the years. Despite multiple visits to different doctors, no treatment was offered, leaving us anxious about his future–fearing pain, bleeding, and restricted movements. When we finally consulted the dermatology department at this hospital, the team conducted a detailed evaluation and reassured us that, although rare, his condition was treatable. The treatment plan, including sclerotherapies, gave us hope. After the procedures, we saw a noticeable reduction in the lesion size and thigh thickness. The doctors have assured us that the procedure was successful. We are truly grateful for their expertise and coordinated approach, which has made such a difference in our child's life. We now look forward for his smooth and speedy recovery. (*Translated from Marathi to English*).

## Data Availability

The original contributions presented in the study are included in the article/Supplementary Material, further inquiries can be directed to the corresponding author.
