# Supplementary figures and images for: Multi-disciplinary diagnosis and management of verrucous venous malformation of the right knee: a case report
Source: Front Radiol. 2026 Jan 13;5:1686404. doi: 10.3389/fradi.2025.1686404 (PMC12834784; doi:10.3389/fradi.2025.1686404)

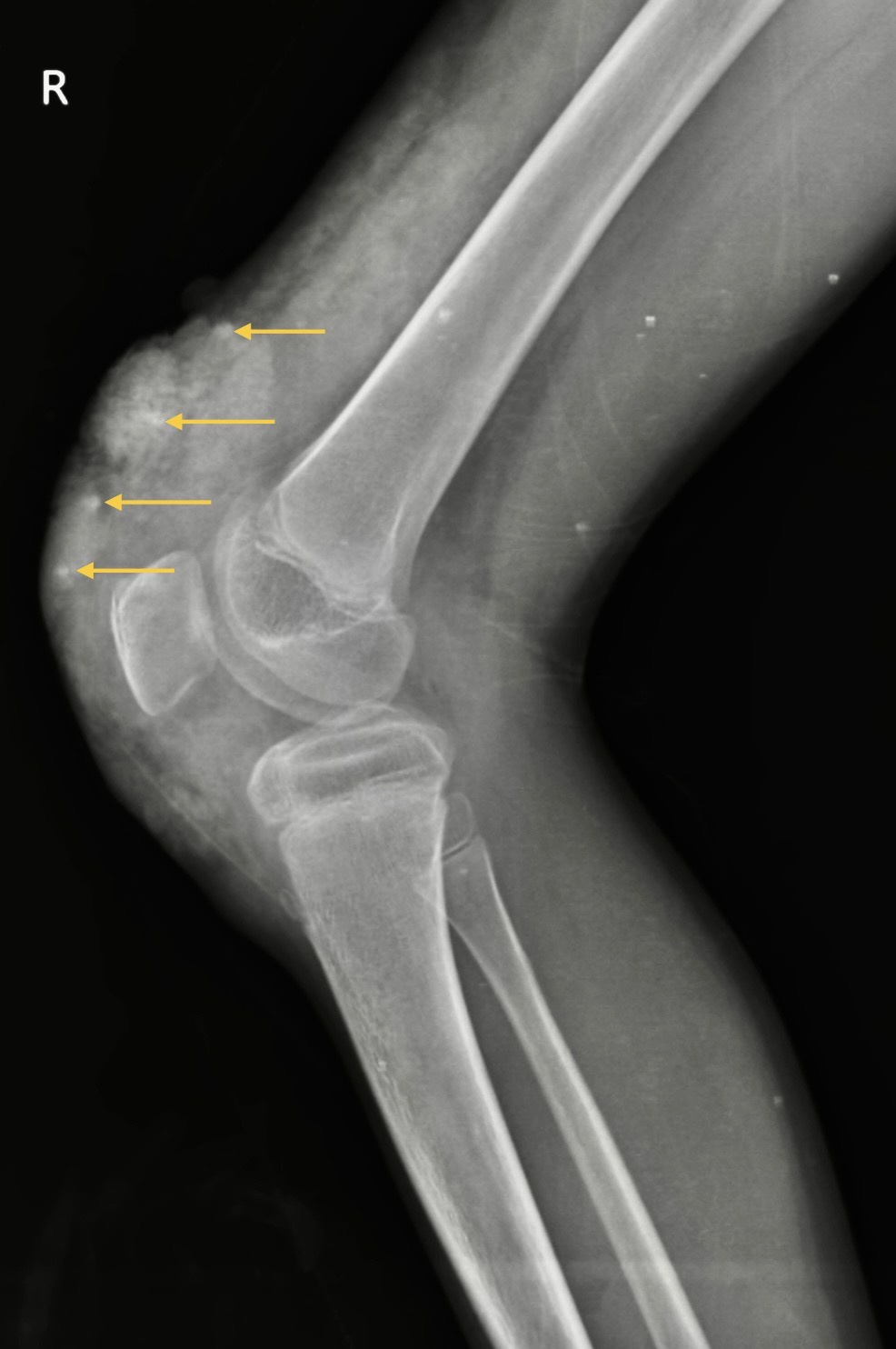

Supplement: Supplementary file 4 [file Image1.jpeg]

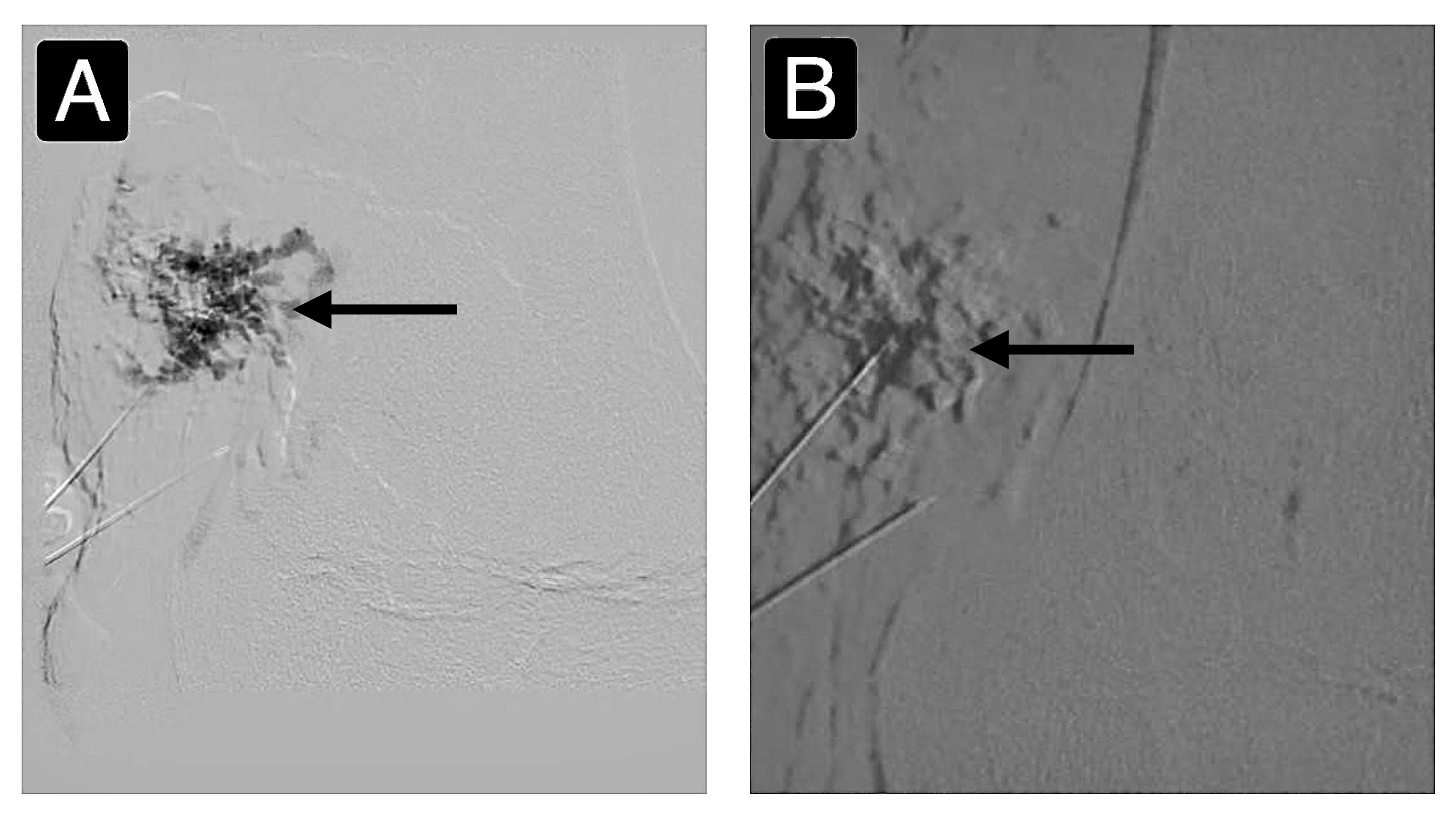

Supplement: Supplementary file 5 [file Image2.jpeg]

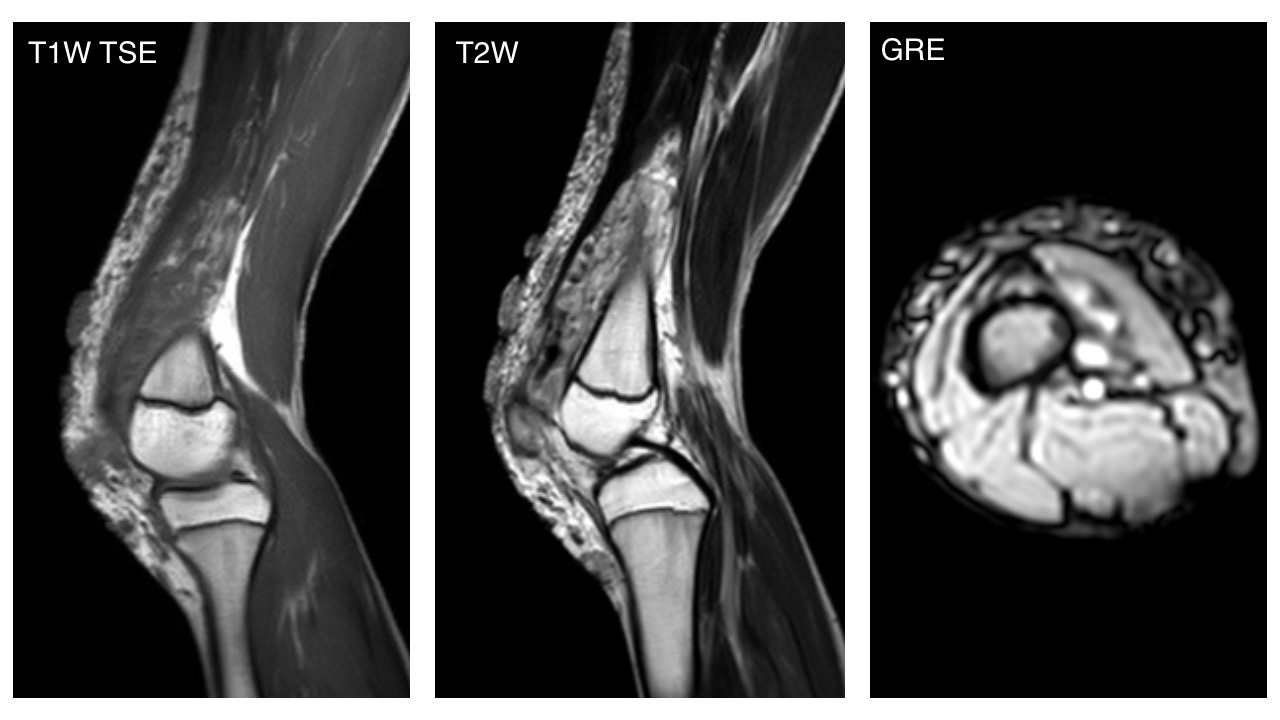

Supplement: Supplementary file 6 [file Image3.jpeg]

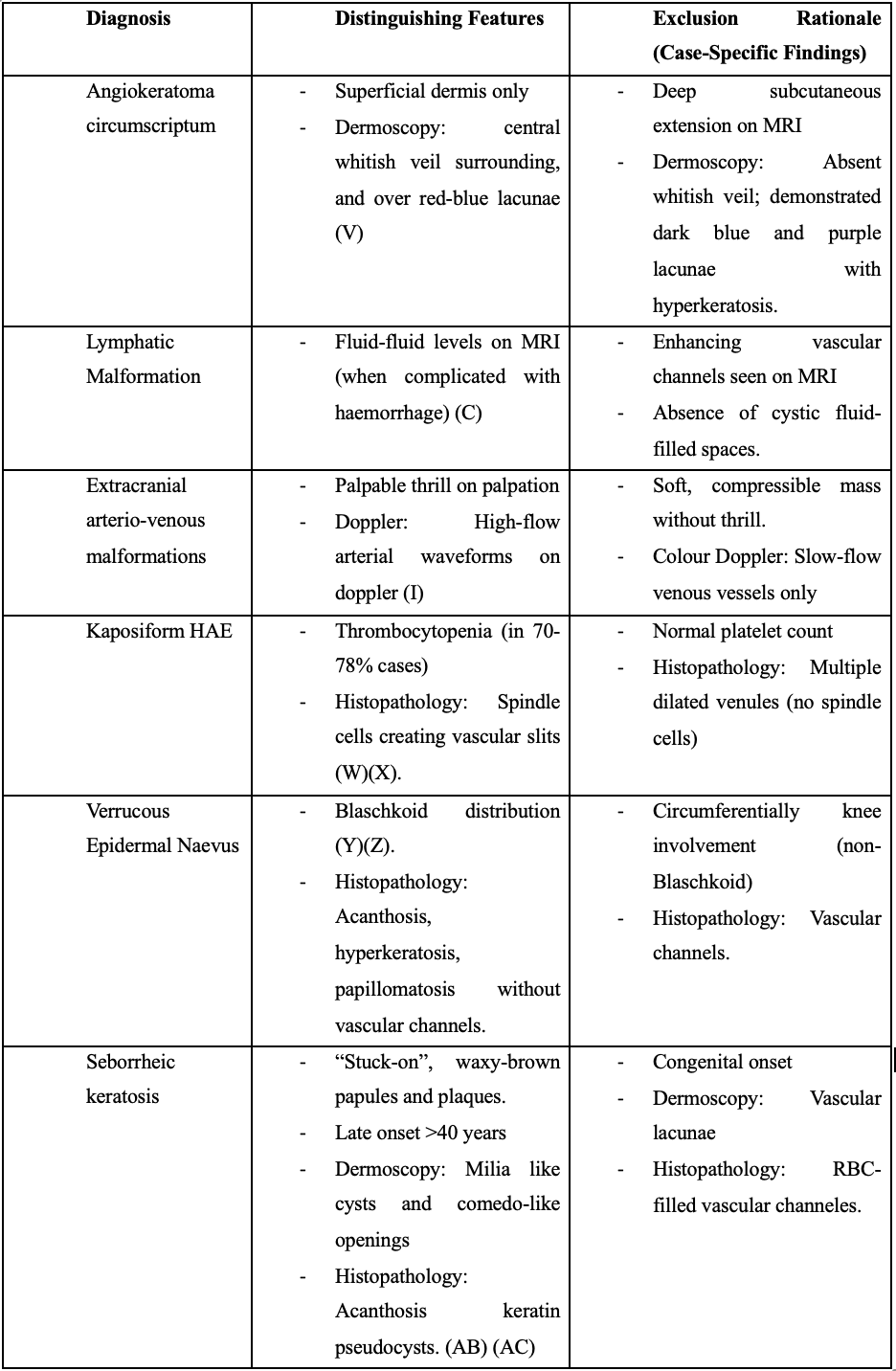

Supplement: Supplementary file 7 [file Image4.png]

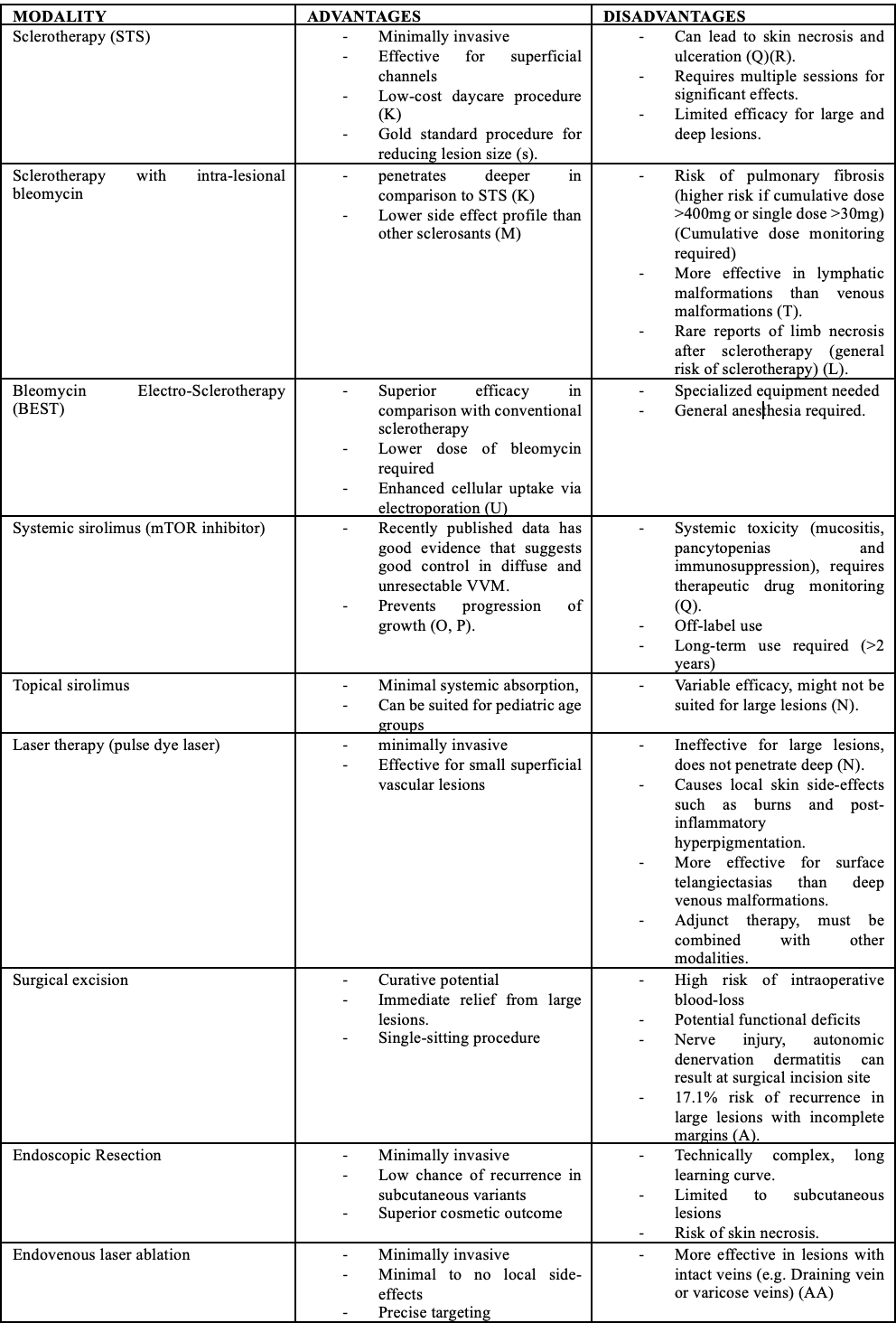

Supplement: Supplementary file 8 [file Image5.png]
